# Effect of Different Phosphate Glass Compositions on the Process-Induced Macromolecular Dynamics of Polyamide 66

**DOI:** 10.3390/polym12051179

**Published:** 2020-05-21

**Authors:** Imane Belyamani, Mohammad K. Hassan

**Affiliations:** 1 Department of Polymer Science and Engineering, The University of Southern Mississippi, 118 College Drive #5050, Hattiesburg, MS 39406, USA; 2 Institut Supérieur de Plasturgie d’Alençon (ISPA), Campus d’Alençon, 61250 Damigny, France; 3 Center for Advanced Materials, Qatar University, Doha P.O. Box 2713, Qatar

**Keywords:** broadband dielectric spectroscopy, polyamide 66, inorganic phosphate glass, chemical mechanism, molecular chain dynamics

## Abstract

The present study provides a fundamental understanding of the mechanism of action of special new phosphate glass (P-glass) systems, having different glass transition temperatures (*T*_g_), in polyamide 66 (PA66). Dynamic mechanical analysis (DMA) revealed that the *T*_g_ of PA66/low *T*_g_ P-glass (ILT-1) was significantly shifted to a lower *T*_g_ (65 °C), and another transition appeared at high temperature (166 °C). This was supported by a drop in the melting point and the crystallinity of the PA66/ILT-1 hybrid material as detected by differential scanning calorimetry (DSC). The dielectric spectroscopic investigation on the networks’ molecular level structural variations (*T*_g_ and sub-*T*_g_ relaxations) agreed very well with the DMA and DSC findings. Contrary to intermediate *T*_g_(IIT-3) and high *T*_g_ P-glass (IHT-1) based materials, the PA66/ILT-1 hybrid material showed an evidence of splitting the PA66 *T*_g_ relaxations into two peaks, thus confirming a strong interaction between PA66 and ILT-1 (low *T*_g_ P-glass). Nevertheless, the three different P-glass compositions did not show any effect on the PA66 sub-*T*_g_ relaxations (related to the –NH_2_ and –OH chain end groups’ motion).

## 1. Introduction

Inorganic phosphate-based glass (P-glass) systems have gained increasing interest in the last decade because of their wide variety of applications and tunable properties [1]. As a consequence, P-glass materials display a wide range of unique features such as chemical durability and stability, good electrical conductivity, excellent optical properties [2], biocompatibility, efficient drug delivery [3,4], and flame-retardancy [5]. The ability to control the chemical composition of P-glasses to suit the target application requirements makes them desirable additives for organic/inorganic hybrid materials.

The potential of polymer/P-glass materials lies behind the ease to tailor the glass transition temperature (*T*_g_) of P-glass [1] to make them fluidic over a broad range of temperatures in which many polymers melt. This property allows melt processing polymer/P-glass hybrids using conventional polymer processing techniques such as extrusion and injection molding.

Since P-glasses are a new evolving class of materials, only a few research groups have investigated the impact of P-glass on the final properties of P-glass/polymer, particularly P-glass/polyamide materials [5,6,7,8]. For instance, it has been shown that for tin fluorophosphate glass (TFPg)/polyamide 6 (PA6) material containing ≥5 vol % TFPg, the strain–stress curve is remarkably consistent with that of typical plasticized polymers with a drop in the Young modulus and an increased elongation at break [6]. More recently, Belyamani et al. [5], have demonstrated that 15 wt % of TFPg decreased both the peak heat release rate and the total heat amount released from the TFPg/polyamide 66 (PA66) hybrid materials. Nevertheless, the mechanism of action of P-glass at the molecular-level length scales and the dynamics of P-glass/polyamide hybrids are still not fully understood. Very few attempts to understand the exact mechanism by which these inorganic molecules act in polyamides have been suggested in the literature [7,9,10,11,12,13]. In 2006, Urman and Otaigbe [7] provided the first proof of a partial miscibility between TFPg and PA6, leading to the melting point and *T*_g_ depression of the binary material after the addition of 10 vol % TFPg. The observed melt miscibility was attributed to potential physicochemical interactions at the interfacial layers between PA6 chains and TFPg [7,9]. This theory was confirmed later by Urman et al. through the relaxation behavior of P-glass/PA6 hybrids using broadband dielectric relaxation spectroscopy [13].

Broadband dielectric spectroscopy (BDS) is considered by many researchers to be one of the most useful techniques for evaluating the dynamics of polymeric materials and is widely used for that purpose [14,15]. A dielectric response results from the interaction of dipoles or polarizable elements with an applied electric field that oscillates at different frequencies (*f*) at given temperatures. The imaginary part, ε″, or loss permittivity, of this response, is proportional to the energy dissipated per cycle during any of the polymer relaxation processes. These relaxations include the short-range and long-range cooperative motions, which take place on extremely different time scales, can be precisely examined vs. temperature, and over a broad frequency range that extends from 10^−3^ to 10^7^ Hz region.

Several studies in the literature have reported the relevance of BDS to examine the miscibility degree and the interfacial interactions in both organic/inorganic and organic/organic materials. However, apart from Urman et al. [13] research work on low *T*_g_ P-glass/PA6 material, to the best of our knowledge, no study has been conducted to provide a clear fundamental understanding on the molecular dynamics of PA66 matrix consequent to the addition of different P-glass compositions and *T*_g_.

The present work follows up on our previous study [5] as to the flame retardant efficiency of special new inorganic phosphate glass (P-glass) compositions, having different *T*_g_ in PA66. In this mentioned article, the low *T*_g_ P-glass composition (i.e., ILT-1) was shown to exhibit a promising flame retardant behavior in PA66 at a concentration of up to 15 wt % compared to intermediate (IIT-3) and high (IHT-1) *T*_g_ P-glasses; ILT-1 decreased both the peak heat release rate and the total heat amount released from the PA66/ILT-1 hybrid materials as a result of an efficient glassy char layer formation. These intriguing findings prompted us to address several questions concerning the molecular dynamics of P-glass/PA66 as a function of the different studied P-glass systems.

From a flame-retardant application perspective, the mechanism of action of phosphorus-based flame retardant additives occurs mainly during the combustion stage [16,17,18,19,20,21]. Nonetheless, the present work provides evidence that the P-glass mode of action in PA66 happens during the melt processing operations. Thermal analysis, along with broadband dielectric spectroscopy experiments, was used to investigate the changes in the phase transitions of PA66 consequent to P-glass addition during the melting process. It is worth noting that all tests were conducted on processed materials without any further treatment.

## 2. Materials and Methods

### 2.1. Materials

DuPont™ (Wilmington, DE, USA) provided the PA66 (Zytel^®^ 101) used in the present work. Before processing step, PA66 was dried at 80 ± 1 °C under a vacuum of 28 ± 1 inch Hg until a water content of 0.05 wt % was reached.

P-glass with 3 different glass transition temperatures (*T*_g_) were synthesized in the laboratory, as reported previously by us [5]. The tin fluorophosphate glass composition (hereinafter denoted as ILT-1) results in a P-glass with a *T*_g_ of approximately 104 °C. The mixed alkali zinc phosphate glasses with an intermediate *T*_g_ (hereinafter denoted as IIT-3) and a high *T*_g_ (hereinafter denoted as IHT-1) have respectively glass transition temperatures of 192 °C and 314 °C.

### 2.2. Melt Processing and Characterization Methods

#### 2.2.1. Internal Batch Mixing

Processing operations were carried out on dry PA66 (0.05% water content), using a thermos-Haake Polydrive mixer (Thermo Fisher Scientific, Waltham, MA, USA), at a temperature of 263 °C (Zones 1, 2, and 3). Three formulations were performed at 15 wt % P-glass and for the different P-glass compositions. First, the polymer was added to the mixing bowl (time 0) and allowed to mix for 2.5 min to yield a homogeneous melt. P-glass was then introduced to the mixer and allowed to mix for an additional 2.5 min. The rotor speed was fixed at 75 rpm. Neat PA66 was processed at the same conditions as a control for all characterization measurements.

#### 2.2.2. Differential Scanning Calorimetry (DSC)

The measurements were performed on approximately 12 mg samples using a Perkin Elmer differential scanning calorimetry (DSC; Perkin Elmer, Waltham, MA, USA). Samples were equilibrated at 150 °C, ramped at 10 °C/min to 290 °C, cooled at 10 °C/min to 150 °C, and reheated at 10 °C/min to 290 °C. The reported data represent the melting temperature (*T*_m_) of the second heating cycle.

#### 2.2.3. Dynamic Mechanical Analysis (DMA)

Dynamic storage modulus E′ and loss factor tanδ = *E*″/*E*′ (*E*″ is loss modulus) vs. temperature curves were generated using a DMA Thermal Analysis Q800 instrument (TA Instruments, New Castle, DE, USA). The instrument was operated in a tensile mode at a frequency of 1 Hz and a strain of 0.028%. All experiments were carried out over the temperature range −20 to 220 °C at a heating rate of 3 °C/min.

#### 2.2.4. Tensile Strength

Stress vs. strain measurements were conducted using a material testing system (MTS) 810 test-frame (MTS Systems Corporation, Eden Prairie, MN, USA) and analyzed using an MTS Testworks 4.0 software package. The strain rate was set at 10 mm/min for all experiments. Five injection-molded dog-bone specimens ASTM D638/Type I were tested for each material and the obtained average values are reported.

#### 2.2.5. Compressing Molding

Dielectric samples were prepared by compression molding the PA66/P-glass hybrids and the processed neat PA66 into 30 µm films following standard methods. The compression molding conditions were set as follows: temperature = 272 °C, pressure = 250 MPa, and mold residence time = 2 min.

#### 2.2.6. Broadband Dielectric Spectroscopy (BDS) Measurements

Dielectric spectra were collected isothermally using a Novocontrol GmbH Concept 80 Broadband Dielectric Spectrometer (Novocontrol Technologies GmbH, Montabaur, Germany) over the frequency range 0.1 Hz–3 MHz and temperature range −60–200 °C. Temperature stability was controlled to within ±0.2 °C. Samples were cut to a diameter of 2 cm and placed between two 2 cm diameter gold-coated copper electrodes. Aluminum foil was sandwiched between the specimen and the electrode to prevent the polymer from adhering to the gold coating of the electrode. Verification tests were conducted to ensure that the aluminum foil did not alter the dielectric spectra of PA66 and hybrids. Electrode-sample assemblies were then transferred to the instrument for data collection. Three replicates per each sample were conducted.

## 3. Results and Discussion

### 3.1. Differential Scanning Calorimetry (DSC) Analysis

**Figure 1:**
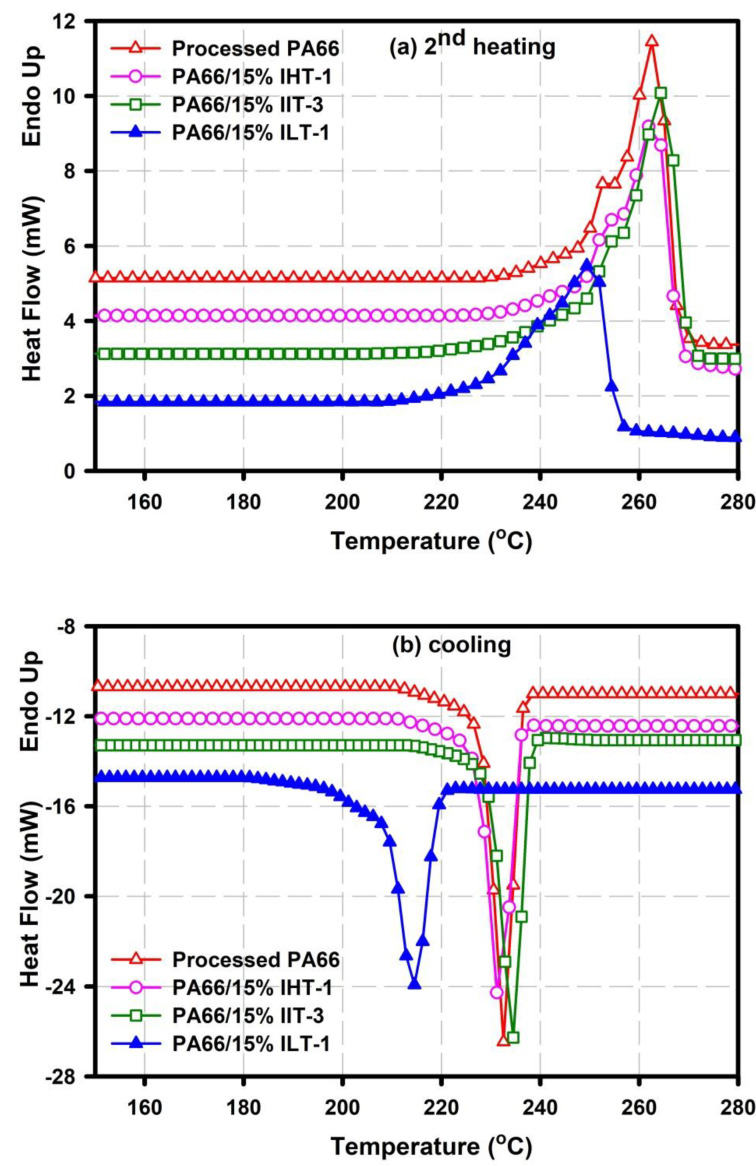
(**a**) 2^nd^ heating, and (**b**) cooling differential scanning calorimetry (DSC) thermogram for the studied PA66/P-glass systems and processed virgin PA66.

Figure 1 shows the 2^nd^ heating and the cooling DSC thermogram of the investigated hybrids, as compared to those of the neat PA66, and Table 1 summarizes their thermal properties. DSC results of PA66/IHT-1 and PA66/IIT-3 samples display an endothermic melting peak around 263 °C and an exothermic one at 232 °C, similar to those observed for the neat PA66 matrix. However, when blended with ILT-1 P-glass, PA66 melting and crystallization points shift to a lower temperature (250 and 215 °C, respectively), indicating a change in its crystallization.

Furthermore, using Equation (1), the crystallinity (*X*_c_) of PA66 component in the PA66/ILT-1 system was found to significantly decrease (*X*_c_ = 15.05%) upon the addition of ILT-1 P-glass as compared to that of the neat matrix (*X*_c_ = 34.1%), as it is depicted in Table 1.

(1) $$X_{c}=\Delta H_{m}/\left(\Delta H_{0}\times w\right)$$

where Δ*H*_0_ is the melting enthalpy of 100% crystalline PA66 (197 J/g) [22], Δ*H*_m_ is the apparent melting enthalpy corresponding to the PA66/P-glass hybrids, and *w* is the weight fraction of PA66.

It has been demonstrated that in blends having crystallizable components, depression of both melting point and crystallinity, as well as the crystallization delay, are indicative of the miscibility in the amorphous state [23]. Additionally, the thermodynamically favorable interaction between blended phases and/or the decrease of chain mobility resulting from crosslinking reactions reflect the same phenomena [24,25].

To further explore the extent of a potential chemical reaction between PA66 and ILT-1, dynamic mechanical analysis was performed.

### 3.2. Dynamic Mechanical Analysis (DMA)

**Figure 2:**
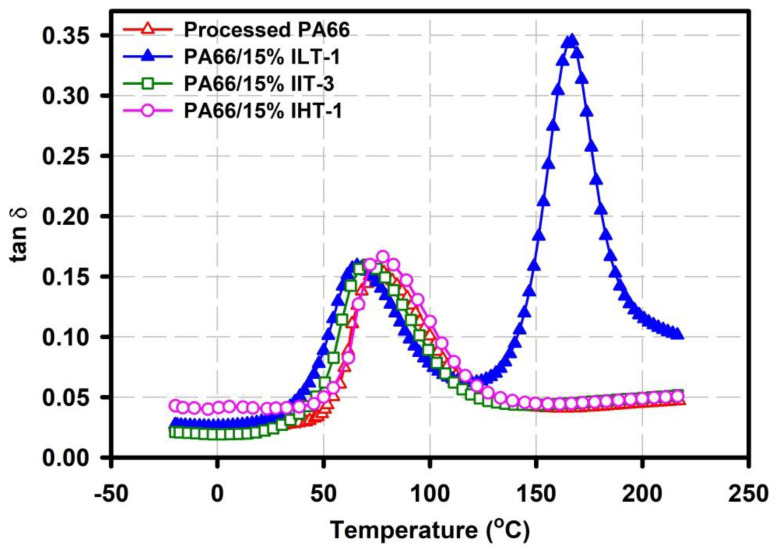
Loss factor tanδ curves as a function of temperature for the studied PA66/P-glass systems and processed virgin PA66.

DMA is a useful tool for the interfacial interaction characterization of filled polymer systems. Figure 2 shows the tanδ of virgin processed PA66 resin and PA66/ILT-1, PA66/IIT-3, and PA66/IHT-1 hybrid materials as a function of temperature. tanδ is plotted against temperature and glass transition is typically observed as a peak since the material will absorb energy as it passes through the glass transition.

It is seen in Figure 2 that the tanδ curves of virgin PA66, PA66/IIT-3, and PA66/IHT-1 networks display one unique and strong relaxation corresponding to their glass transitions, labeled α. Moreover, the addition of IHT-1 and IIT-3 P-glass insignificantly shifts the PA66 *T*_g_ (75 °C) to 78 and 70 °C respectively. In contrast to IIT-3 and IHT-1, the addition of ILT-1 considerably shifts the PA66 *T*_g_ to a lower temperature (65 °C) and gives rise to another transition at 166 °C, indicating the presence of another crystalline phase in PA66.

**Figure 3:**
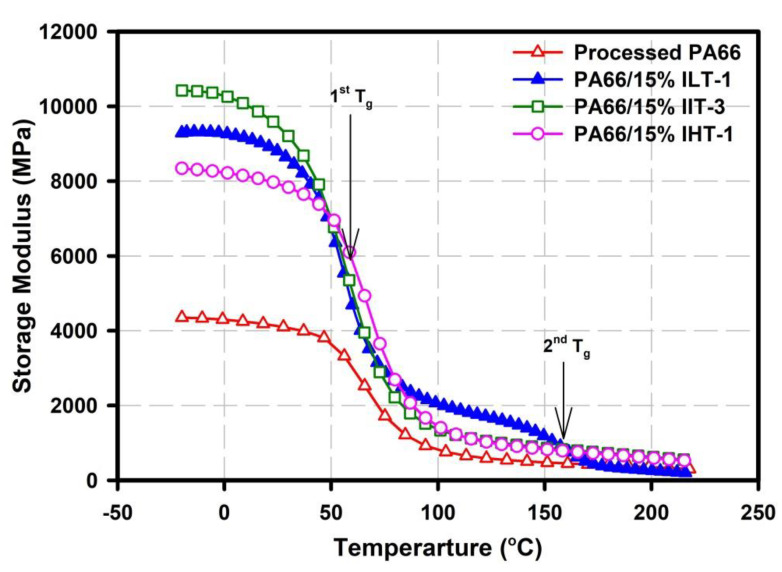
Storage modulus curves as a function of temperature for the studied PA66/P-glass systems and processed virgin PA66.

Values of storage modulus, describing the stiffness of the material, were also plotted as a function of temperature for all studied samples, as depicted in Figure 3. It is observed that the glassy storage modulus of PA66 increased 2–3 times with the addition of P-glass additives regardless of their *T*_g_, suggesting a reinforcing effect on the dynamic modulus.

Additionally, except the PA66/ILT-1 formulation, all storage modulus curves displayed in Figure 3, showed three distinct regions: a glassy high modulus region where the segmental mobility is restricted, a transition zone where a substantial decrease in the modulus values with the increase of temperature, and a rubbery region where a drastic decay in the modulus with temperature was observed. PA66/ILT-1 sample shows a similar trend, nevertheless, a 2^nd^ transition at about 166 °C was observed, thus confirming the tanδ vs. temperature data.

**Figure 4:**
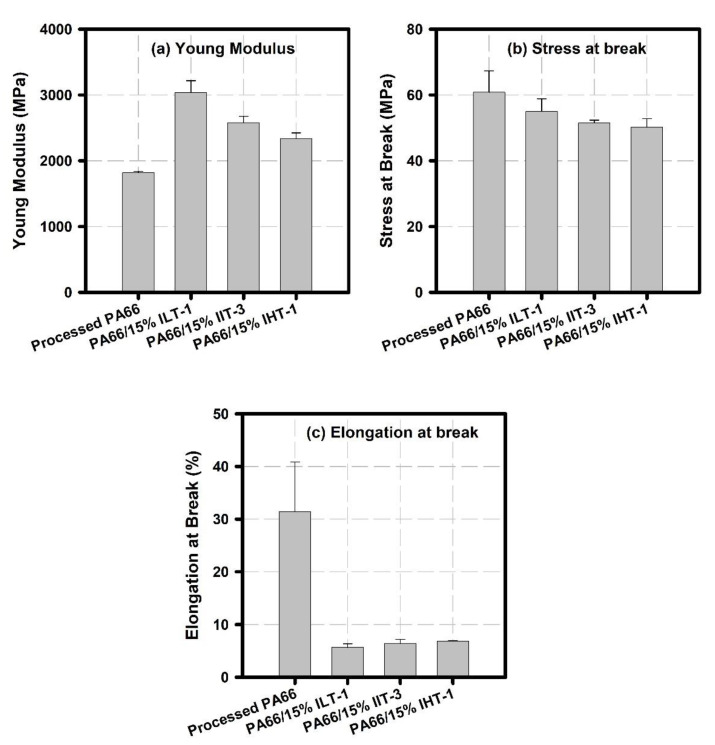
(**a**) Tensile modulus, (**b**) stress at break, and (**c**) elongation at break for the studied PA66/P-glass systems and processed virgin PA66.

To further understand the DMA findings, mechanical tensile experiments at room temperature were conducted, and the obtained results are illustrated in Figure 4. A significant increase in the elastic modulus was observed for all hybrids and, in particular, for PA66/ILT-1 sample, which exhibited a tensile modulus twice as high comparing to the virgin PA66. This result is in a strong agreement with the big increase in storage moduli for the hybrids before the *T*_g_ phase (Figure 3). While the stress at break was not significantly impacted by P-glass introduction, a significant drop of the PA66/P-glass elongation at break was observed.

From DSC, DMA, and mechanical tensile findings, two different scenarios can be made:

The observed thermal and mechanical behavior of PA66/ILT-1 formulation may arise from the presence of degraded PA66 segments and PA66—ILT-1 crosslinked chains. The obtained crosslinking residues increased polymer–polymer interactions and decreased free volume, which in turn reduced their mobility. Consequently, PA66/ILT-1 samples significantly became stronger and stretched less than virgin PA66 samples.

According to the discussed behavior of intermediate and high *T*_g_ P-glasses and our previously published results [5], IIT-3 and IHT-1 systems did not show any evidence of a chemical reaction with PA66. Thus, it can be assumed that IIT-3 and IHT-1 act as a reinforcing filler for PA66.

These stated assumptions are also supported by the rheological data reported by Urman and Otaigbe [6] on low *T*_g_ P-glass/PA12 systems. The authors observed a dramatic decrease in PA12 viscosity upon the addition of >2 vol % of P-glass resulting from potential interfacial interactions between PA12 and P-glass phases.

For further insight into the effect of ILT-1 P-glass in PA66, broadband dielectric spectroscopy (BDS) measurements were carried out.

### 3.3. Dielectric Spectroscopy Analysis

The BDS analysis of the networks’ molecular level structural variations (i.e., hybrids chain motion, *T*_g_, and sub-*T*_g_ relaxations) is a powerful tool of interrogation; the information can be collected over a broad frequency (*f*) range so that motional processes that occur over broad time and distance scales can be investigated vs. temperature.

#### 3.3.1. γ relaxation

**Figure 5:**
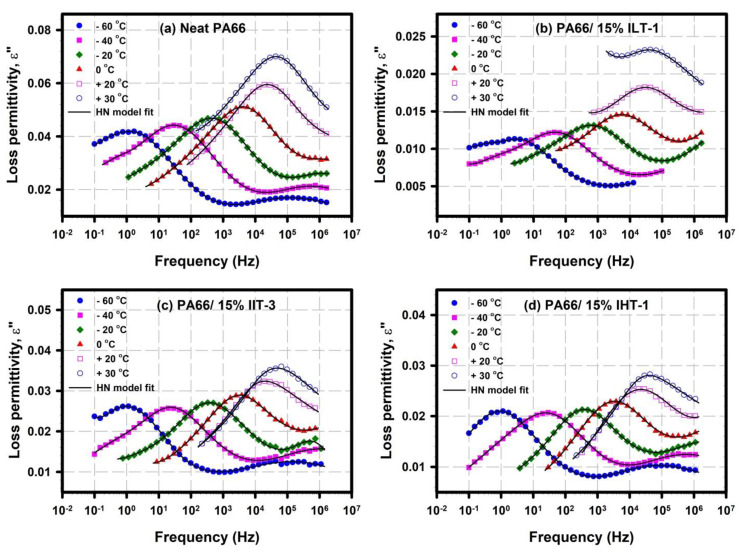
ε″ vs. *f* spectra at different temperatures for the studied PA66/P-glass systems and processed virgin PA66 showing the γ relaxation. Lines show the Havriliak–Negami (HN) equation fits to the spectra.

While the *T*_g_ Relaxation is of great importance in studying polymer/hybrid networks, understanding the secondary transitions and their molecular motions are essential for evaluating the mechanical performance of these systems. Figure 5 displays the loss permittivity (ε″) vs. frequency (*f*) spectra of the studied PA66/P-glass systems as compared to the processed virgin PA66 and within the temperature range of the γ relaxation process. The low temperature γ relaxation involves the motion of very short –CH_2_ segments and an amide group, which provides the dielectric activity [26]. It is clearly seen that the loss permittivity ε″ peak maxima for the γ relaxation shift to higher frequencies as the temperature increases, reflecting faster molecular motions and shorter relaxation times in the usual sense.

The widely used Havriliak–Negami (HN) Equation (2) was fitted to the data in Figure 5 to extract more in-depth information related to the dynamics of the *T*_g_ and the secondary γ relaxations of PA66/P-glass systems.

(2) $$\varepsilon^{*}\left(\omega \right)=\varepsilon^{\prime }-i\varepsilon^{\prime \prime }=-i{\left(\frac{\sigma_{dc}}{\varepsilon_{0}\omega }\right)}^{N}+\overset{3}{\underset{k=1}{\sum }}\left[\frac{\Delta \varepsilon_{k}}{{\left(1+{\left(i\omega \tau_{HN}\right)}^{\alpha_{k}}\right)}^{\beta_{k}}}+\varepsilon_{\infty k}\right]$$

ε′ and ε″ are the real and imaginary dielectric permittivities, respectively, and i = √ − 1. There are three relaxation terms in the sum, and the term on the left accounts for dc conductivity. ε_0_ is the vacuum permittivity and ω *= 2πf*. For each relaxation term *k*, the dielectric strength ∆ε*_k_ =* (ε*_R_* − ε_ω_)*_k_* is the difference between ε′ at very low and very high frequencies, respectively. σ*_dc_* is the dc conductivity and the exponent *N* characterizes conduction in terms of the nature of charge hopping pathways and charge mobility constraints [27]. α and β characterize the breadth and degree of asymmetry, respectively, of ε″ vs. ω peaks.

**Figure 6:**
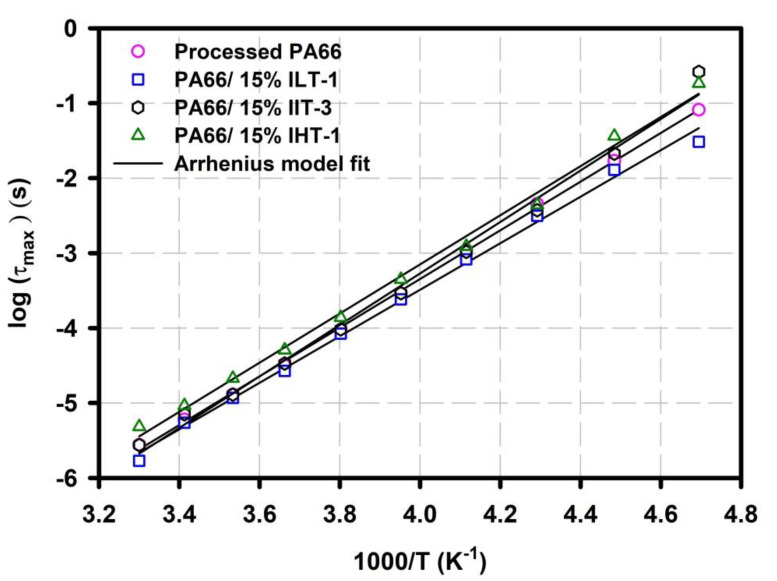
Arrhenius plots of the γ relaxation for the studied PA66/P-glass systems and processed virgin PA66. Solid lines represent the Arrhenius equation fits.

The relaxation time τ_max_ = 1/2 π *f*_max_ was extracted from fitting the data in Figure 5 to the HN equation at each temperature. Log τ*_max_* was then plotted as a function of 1000/T, as shown in Figure 6 depicting a strong Arrhenius behavior according to Equation (3):

(3) $$\tau_{max}\left(T\right)=\tau_{0}\exp\left[E_{a}/\left(RT\right)\right]$$

where *E_a_* is the activation energy of the γ relaxation motion, *R* is the universal gas constant, and τ*_o_* is a pre-exponential factor.

The activation energies, summarized in Table 2, for this molecular motion seem to be similar for neat PA66, PA66/ILT-1, PA66/IIT-3, and PA66/IHT-1 samples, implying the same local γ relaxation motion. Moreover, this energy is in the typical range for secondary relaxations in the glassy state of conventional polymers.

#### 3.3.2. α Relaxation: Glass Transition Chain Dynamics

**Figure 7:**
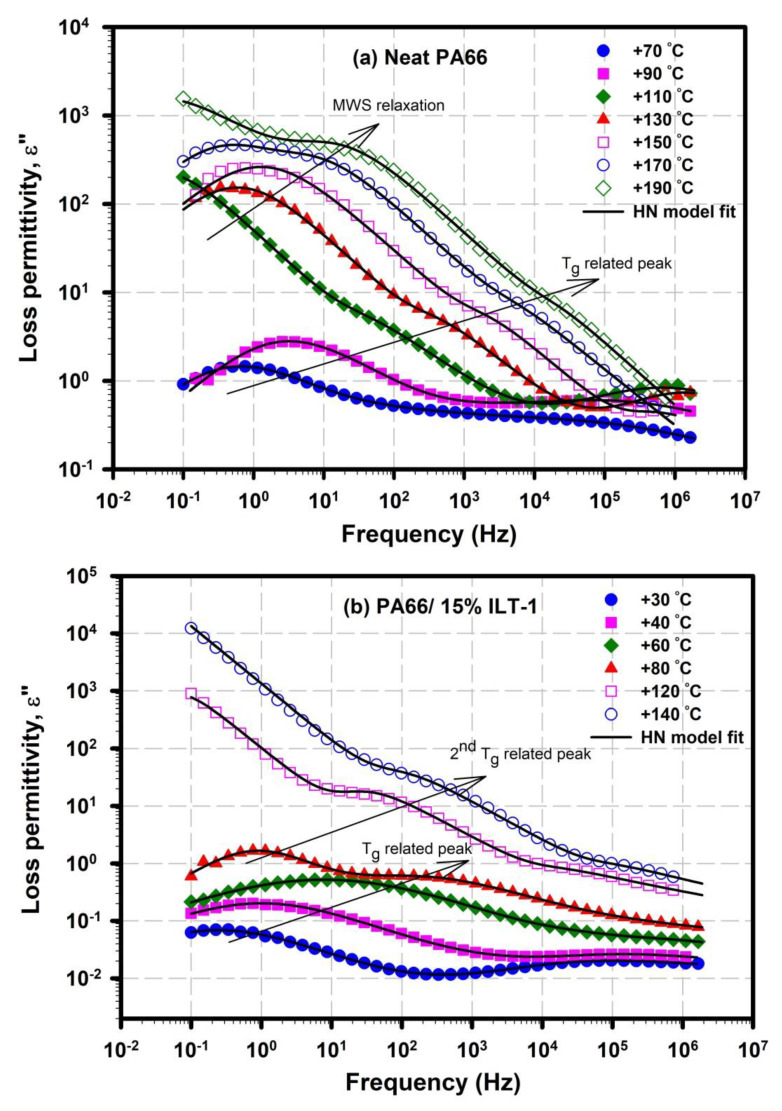
ε″ vs. *f* spectra at different temperatures for the studied PA66/ILT-1sample as compared to the processed virgin PA66 showing the α relaxation. Lines show the HN equation fits to the spectra.

**Figure 8:**
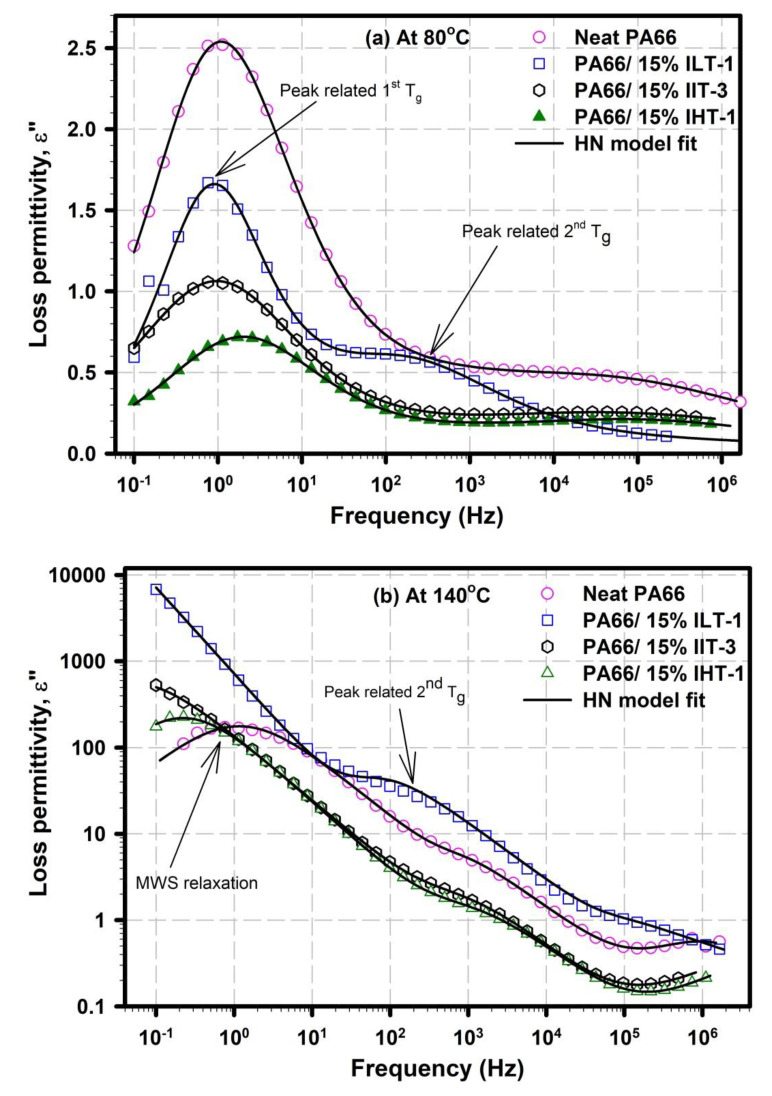
ε″ vs. *f* spectra at (**a**) 80 and (**b**) 140 °C for PA66/P-glass systems and the processed virgin PA66 showing the α relaxation. Lines show the HN equation fits to the spectra.

The α relaxation, or dynamic glass transition, is associated with long-range chain segmental mobility. Figure 7 and Figure 8 display shifts in macromolecular motions related to the glass transition relaxation, and loss permittivity ε″ vs. frequency at 80 and 140 °C spectra, indicating the glass transition-related peaks for each sample, respectively. Figure 7 shows that the *T*_g_ relaxation peak maxima for virgin PA66 and PA66/ILT-1 hybrid samples shift to higher frequencies, indicating slightly faster chain motions as the temperature was increased. Additionally, another relaxation peak appears at 80 °C, which agrees very well with the DMA results depicting an extra peak for the PA66/ILT-1 hybrid sample only.

Another active process detected at low frequency in Figure 7 and Figure 8b for PA66, PA66/IIT-3, and PA66/IHT-1 samples, can be due to the Maxwell–Wagner–Sillars (MWS) interfacial polarization process. This process is common in multiphase systems having different dielectric constants and conductivities among their phases. In contrary to this behavior, the PA66/ILT-1 hybrid does not show similar process, which is possibly owing to the formation of more homogenous network structure.

Once again, the data displayed in Figure 7 and Figure 8 prominently revealed the apparition of a 2^nd^ glass transition temperature for PA66/ILT-1 formulation.

For further investigation on the relaxation processes dynamics elucidated within the *T*_g_ region, the characteristic relaxation times for each relaxation, τ_max_, calculated from the HN equation fitting of the spectra were plotted against the reciprocal of temperature. The Vogel–Fulcher–Tammann–Hesse (VFTH) equation [28,29,30] was then fitted to τ_max_ vs. temperature data for the *T*_g_ relaxation according to Equation (4):

(4) $$\;\tau_{max}=\tau_{0}\exp\left(\frac{E_{a}}{\left[k_{B}\left(T-T_{V}\right)\right]}\right)$$

where *k_B_* is the Boltzmann constant, *τ_0_* is a hypothetical relaxation time at infinite temperature, *E_a_* is the apparent activation energy for the motion, and *T*_V_ is the Vogel temperature. The Vogel temperature is considered as the static freezing temperature [28,29] at which chain segments become frozen.

**Figure 9:**
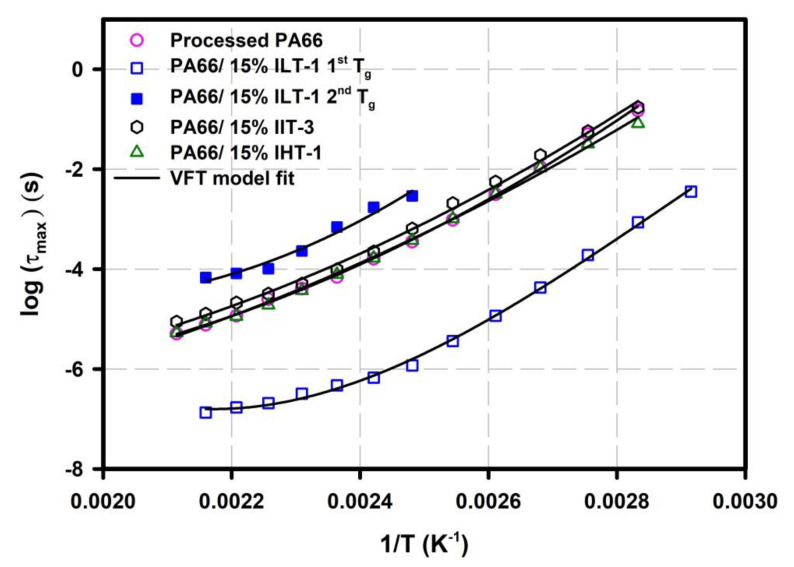
The characteristic relaxation times of the studied PA66/P-glass systems and processed virgin PA66 as a function of temperature. Solid lines represent the VFTH equation fits.

Figure 9 shows the characteristic relaxation time as a function of reciprocal temperature for processed PA66 and PA66/P-glass samples. The solid lines represent fits of the data to the VFTH equation, and Table 3 summarizes the obtained fitting parameters. The two plots for the PA66/ILT-1 sample correspond to separate fittings of the two relaxations in the α transition region as already discussed, and the curvature of all plots is characteristic of long-range motions in glass-forming polymers.

As can be detected in Figure 9, the τ_max_ values for neat PA66, PA66/IIT-3, and PA66/IHT-1 samples across the studied broad range of temperature, where the *T*_g_ related motions get activated, are similar. This signifies that the addition of IIT-3 and IHT-1 P-glass additives does not meaningfully affect the molecular motions associated with the *T*_g_ transition in PA66. However, it is evident that the peak related to the 1^st^ glass transition of the PA66/ILT-1 sample (see Figure 8a) was significantly vertically down-shifted, confirming, once again, that the addition of ILT-1 P-glass accelerates the α relaxation of PA66 chains. Additionally, the curve for the 2^nd^ glass transition in the PA66/ILT-1 sample shows an opposite behavior, reflecting a slower and more restricted chain motions. VFTH fitting parameters, displayed in Table 3, confirm the just mentioned observations; the PA66/ILT-1 1^st^ *T*_g_ related segment relaxation shows the highest *E*_a_ (0.8799 eV) and the lowest *T*_v_ (90.50 K), and PA66/ILT-1 2^nd^ *T*_g_ related segment relaxation shows the lowest *E*_a_ (0.0229 eV) and the highest *T*_v_ (371.5 K) as compared to neat PA66 and the other PA66/P-glass samples. It should be noted that in polymer blend systems, a decrease of the apparent activation energy is often ascribed to partial miscibility of the neat polymers in the blends [6,31,32], and an increase in the *T*_v_ is attributed to polymer degradation [33]. It should be pointed out that the reduction in the 1^st^ *T*_g_ observed for the studied PA66/15% ILT-1 is more significant than that reported by Urman and Otaigbe [6], who linked the *T*_g_ reduction to phase miscibility in the hybrid. In our case, the miscibility between ILT-1 and PA66 matrix seems to be more pronounced due to the much more downward shift in the VFTH curves of PA66/ILT-1 1^st^ *T*_g_ compared to virginPA66. This result was supported by our previous work [5], in which we demonstrated that ILT-1 tended to disperse better than IIT-3 and IHT-1.

From all findings described in the present work, it can be concluded that the PA66/ILT-1 hybrid system is essentially a mixture of small degraded PA66segments and long PA66—ILT-1 crosslinked chains (degradation of PA66 chains, during the processing stage, in PA66/ILT-1 formulation, was previously demonstrated by our team using gel permeation chromatography analysis [5]). The crosslinked and degraded chain segments do not have the same degree of mobility; the mobility of crosslinked PA66—ILT-1 chain is restrained, resulting in longer relaxation times and, therefore, higher *T*_g_ (Figure 9). At the opposite, the disentangled degraded PA66 chain segments are free to relax completely, which results in a lower relaxation time and a shift towards lower *T*_g_.

**Scheme 1:**
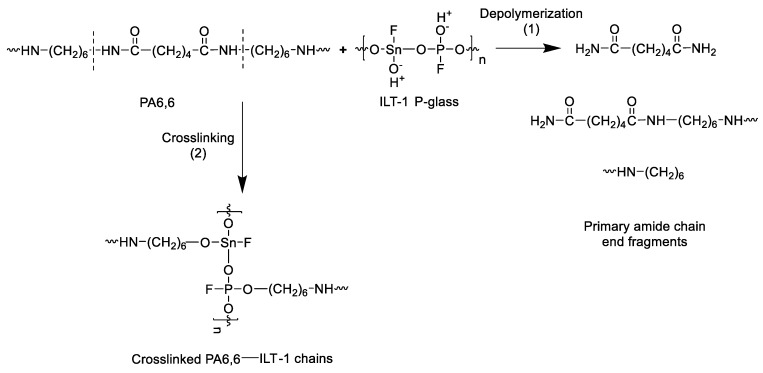
Hypothesized mechanism of action of ILT-1 in PA66 during the melt processing stage.

Based on the present investigations and the reported mechanism of ammonium polyphosphate in PA66 [16], we believe that the addition of ILT-1 to PA66 during melt processing leads to simultaneous depolymerization and crosslinking reactions according to the mechanism shown in Scheme 1.

At 263 °C (processing temperature), ILT-1 P-glass catalyzes the *N*-alkylamide bond scission, through potential protonation of the carbonyl groups, giving primary amide chain end fragments (reaction 1). Simultaneously with the depolymerization reaction, a crosslinking process occurs according to reaction (2) where ILT-1 P-glass acts as a crosslinking agent between the PA66 degradation products, thereby forming ILT-1—PA66 crosslinked network structure.

## 4. Conclusions

Results obtained from different analytical techniques and described in the present work supported and confirmed our previous findings regarding the interaction between the studied P-glass compositions and PA66. Only the low *T*_g_ P-glass shows evidence of chemical interaction with PA66 during the compounding process through a depolymerization/crosslinking mechanism. Blending ILT-1 with PA66 catalyzes simultaneous scission of PA66 chains and crosslinking reactions leading to an evident mixture of crosslinked and depolymerized primary amide end chain fragments. This was confirmed by the depression of both melting point and crystallinity as well as a splitting of the PA66 *T*_g_ relaxations into two peaks. From a flame-retardant application standpoint, the ILT-1/PA66 crosslinked structure is believed to undergo further modification during combustion at high temperatures that would help to form highly efficient glassy char barrier. Advanced solid-state NMR methods (^1^H–^31^P HARDSHIP) will be addressed in future work to give more insight on the proposed mechanism.

## Figures and Tables

**Table 1:** DSC thermal properties for the studied systems.

Sample	*T*_monset_ (°C)	*T*_m peak_ (°C)	*T*_conset_ (°C)	*T*_c peak_ (°C)	*X*_c_ (%)
Processed neat PA66	230	263	242	232	34.10
PA66/15% ILT-1	214	250	221	215	15.05
PA66/15% IIT-3	227	265	240	234	35.80
PA66/15%IHT-1	230	262	242	230	33.50

**Table 2:** Fit parameters of the Arrhenius equation.

Sample	Activation Energy (kJ/mol)	Fit *R*^2^
Processed neat PA66	62.1	0.99964
PA66/15% ILT-1	59.4	0.99818
PA66/15% IIT-3	65.7	0.99612
PA66/15%IHT-1	62.7	0.99755

**Table 3:** Fit parameters of the VFTH equation.

Sample	τ_0_ (s)	*E*_a_ (eV)	*T*_v_ (K)
Neat PA66	3.082 × 10^−13^	0.4397	161.4
PA66/15% ILT-1 1^st^ *T*_g_	1.000 × 10^−20^	0.8799	90.50
PA66/15% ILT-1 2^nd^ *T*_g_	3.261 × 10^−06^	0.0229	371.5
PA66/15% IIT-3	1.691 × 10^−11^	0.3003	204.0
PA66/15%IHT-1	2.904 × 10^−12^	0.3537	184.8
